# Pulmonary function assessment after COVID-19 in vaccinated healthcare workers

**DOI:** 10.1186/s12995-023-00400-7

**Published:** 2023-12-15

**Authors:** Lorenzo Ippoliti, Luca Coppeta, Giuseppina Somma, Giuseppe Bizzarro, Francesco Borelli, Teresa Crispino, Cristiana Ferrari, Ilaria Iannuzzi, Andrea Mazza, Agostino Paolino, Andrea Magrini, Antonio Pietroiusti

**Affiliations:** 1 Department of Biomedicine and Prevention, University of Rome Tor Vergata, Rome, 00133 Italy; 2 Saint Camillus International University of Health Sciences, Rome, 00131 Italy

**Keywords:** COVID-19, Spirometry, Vaccination, Respiratory function, Healthcare workers, SARS-CoV-2

## Abstract

COVID-19 typically presents with flu-like symptoms due to the viral infection itself. The most severe cases are characterised by lung damage, an important factor in fatal outcome due to alveolar damage. In some cases, patients develop a long COVID with persistent symptoms of chest pain and fatigue. Causes, including organ damage or inflammation, are being investigated. Clinical outcomes are variable and permanent lung damage is not fully understood, while vaccination is effective against severe infection but its effect on respiratory function in mild cases remains uncertain. This retrospective study aims to analyse changes in lung function in HCWs who had COVID-19 between 2020 and 2022, comparing their spirometric test results before and after the pandemic and taking into account their vaccination status. 321 HCWs were included in the study. The study examined spirometric parameters both before and after the pandemic, and all measured outcomes except the FEV1/FVC ratio showed a significant decrease during the study period. We then assessed the association between SARS-CoV-2 infection and changes in lung function parameters, analysing infections in 2020, 2021 and 2022 separately. We found a statistically significant difference in Forced vital capacity (FVC) between infected and non-infected subjects in 2020 and 2021, but not in 2022. To evaluate the protective effect of SARS-CoV-2 vaccination on respiratory function, a linear regression analysis was performed using changes in FVC, Forced expiratory volume in 1 s (FEV1), FVC/FEV1 ratio and Peak expiratory flow (PEF) as dependent variables. The analysis showed that the decline in FVC was significantly lower in subjects who had been vaccinated prior to infection. The study concludes that subclinical SARS-CoV-2 infections in 2020 and 2021 worsened respiratory parameters (FVC and FEV1), but vaccination protected against these effects. Even healthy individuals with previous infections showed respiratory changes, with vaccination providing protection, especially for FVC decline. This highlights the importance of vaccinating healthcare workers against COVID-19.

## Background

Typically, COVID-19 presents with flu-like symptoms that are due to the viral infection itself. Severe cases of COVID-19 are mainly characterised by lung damage, which is the main cause of fatal outcome due to severe alveolar damage. This damage differs from classical respiratory distress syndrome (ARDS) by the absence of symptoms proportional to the severity of hypoxaemia [1]. Information from autopsy studies, including minimally invasive autopsies with transthoracic or transbronchial post-mortem necropsy, has provided insight into the main pathological features, course and progression of lung changes [2]. Gross examination of the lungs typically shows weight gain with oedema and diffuse congestion [3]. Cross-sectional images show irregularly distributed areas of consolidation, and in some cases there are areas of haemorrhage or infarction, often accompanied by visible thrombosis in the feeding vessels [4]. The long-term effects of COVID-19 may be caused by these changes. There is still much uncertainty about pulmonary outcomes in patients treated with COVID-19. The radiological evaluation of a person with a positive history of SARS-CoV-2 infection who presents with dyspnoea after resolution of the acute infection should include a chest CT scan performed in the inspiratory phase with thin-slice and overlapping reconstructions (≤ 1.5 mm) and, if possible, expiratory phase acquisitions [5]. A recent systematic review reported that approximately 40% of subjects with prior COVID-19 had a change in DLCO, 15% had findings suggestive of restrictive pathology, and 7% had obstructive patterns [6]. Some patients develop so-called long COVID, which refers to persistent symptoms that occur within 3 months of SARS-CoV-2 infection and last for at least 3 months without any other identifiable cause [7]. The most common symptoms are chest pain, fatigue, dyspnoea and cough [8]. The exact causes are still under investigation, but could include organ damage, persistent inflammation or an immune response. Changes in respiratory spirometric parameters after COVID-19, which could provide very important information in this regard, have not been well studied in the literature. Furthermore, while SARS-CoV-2 vaccination has been shown to be highly effective in reducing the rates of severe infection, hospitalisation and respiratory complications, the effectiveness of COVID-19 vaccine in preventing subclinical changes in respiratory function following mild breakthrough infection has not been evaluated.

### Objective

In this study, we aimed to assess any variation in pulmonary function parameters in a population of healthcare workers (HCWs) who experienced COVID-19 during the period 2020–2022 by comparing spirometric tests performed before and after the SARS-CoV-2 pandemic, in relation to their vaccination status.

## Methods

### Study design

In this study we retrospectively collected lung function tests of HCWs who underwent their health surveillance visit at the Policlinic of Rome Tor Vergata in the period 2019–2023.

At the policlinic, HCWs undergo an annual spirometric test to assess their fitness for work. In accordance with the recommendations of the Italian Ministry of Health, routine pulmonary function tests were suspended during the SARS-CoV-2 pandemic (from March 2020 to September 2022) due to the high risk of SARS-CoV-2 transmission.

To assess any changes in respiratory function that could be attributed to SARS-CoV-2 infection, we compared spirometry tests collected in the six months prior to March 2020 with those performed by the same operators in the six months following September 2022.

### Data collection

Data on SARS-CoV-2 infections were collected from the occupational health database. During the pandemic, each hospital HCWs had to undergo a nasopharyngeal swab every 15 days, and the results of these tests were collected in a unique database. In addition, symptomatic SARS-CoV-2 infections occurring in those operators were routinely reported to the occupational health department and recorded in the same database. Thus, data on both symptomatic and asymptomatic COVID-19 were available for all hospital workers. SARS-CoV-2 vaccination history was extracted by the regional vaccine database that includes all residents who received the Sars-CoV-2 vaccine in the period 2020–2023.

### Spirometry test

Spirometric test was conducted accordind to ERS/ATS standards [9]. Forced vital capacity (FVC), forced expiratory volume in one second (FEV1), FEV1/FVC ratio (Tiffeany index), and peak expiratory flow rate (PEFR) were measured with a spirometer calibrated daily by the same technician according to ATS criteria. The three best measurements of FEV1 and FVC were selected.

### Inclusion and exclusion criteria

HCWs had to meet the following criteria to be included in the study: having had a valid pulmonary function test both before and after the pandemic (in the previously described defined period), absence of significant comorbidities, mainly respiratory diseases, availability of results from molecular swab tests performed during the pandemic period, availability of documentation on vaccination status. Subjects with severe infection were excluded from the study (respiratory failure, septic shock and/or multiple organ dysfunction or failure).

### Data analysis

The following parameters were collected for each partecipant: gender, age, weight and height (BMI), smoking habit, comobridities, COVID-19 (number of episodes and date), and vaccination for SARS-CoV-2 (number of doses). Results of spirometric tests both in the pre- and in the post-pandemic period were also collected and the following paramethers were recorded: FVC, FEV1, Tiffenau index and PEF. All spirometry was recorded as a measured value (lt for volumes and l/sec for flows) and as a percentage of the predicted value.

Data were entered into an Excel database for subsequent analysis. Mean, standard deviation, range, and distribution were calculated for continuous variables. Categorical variables were expressed in terms of frequency.

A paired sample t-test analysis were conducted to analyze spirometric parameters in the pre- and in the post-pandemic period. A linear regression was also used to compare the year of disease onset with each spirometric parameter.

Subsequently, linear regression was performed between the examined variables (FVC, FEV1, Tiffenau index and PEF, expressed as a percentage reduction in mL compared to the period before the pandemic) and the investigated factors (age, BMI, gender, current smoking habit, SARS-CoV-2 infection, prior vaccination, number of infections) for multivariate analysis.

For association tests with the listed variables, a significance level of 5% (p < 0.05) was required.

## Results

We analyzed data from 321 HCWs. Five subjects were excluded from the study. One had a severe infection and the other four had respiratory comorbidities (asthma and COPD). Mean age was 48,85 ± 10,63 (range 20–74) years. Regarding COVID-19, 55.1% of the subjects (177) had a recognized infection in the examined period. Concerning vaccination history, 95.0% (305) of the study population received the vaccination for SARS-CoV-2, and of these 40.7% (124) developed a breakthrought infection after receiving at least one dose of vaccine. The main characteristics of the study population are shown in Table 1.

**Table 1:** Main characteristics of the study population

		n	%	S.D.
Total		321		
Gender	Male	139	43,3	
	Female	182	56,7	
Age (mean)		48,85		10.63
BMI (mean)		24,09		4.01
Smokers	No	188	58.6	
	Ex	64	19.9	
	Yes	69	21.5	
COVID-19	No	144	44.9	
	Yes	177	55.1	
Year of infection	2020	38	21.5*	
	2021	21	11.8*	
	2022	145	81.2*	

S.D. = Standard Deviation

*% related to subjects with COVID-19

Table 2 provides data on the vaccination status of subjects in relation to COVID-19.

**Table 2:** COVID19 and vaccination status

			n	%
COVID-19	Yes		177	55,1
		Vaccinated	124	38.6
		Not Vaccinated*	53	16.5
	No		144	44,9
		Vaccinated	136	42.4
		Not Vaccinated	8	2.5

*Not Vaccinated before the infection

Spirometric paramethers were analyzed both in the pre- and in the post-pandemic period. All collected outcome, except FEV1/FVC ratio showed a significant reduction during the study period when tested at paired-samples t-test (Table 3).

**Table 3:** Main lung function parameters in the spirometry pre and post pandemic period

		Mean	Difference	S.D.	S.E.	95% C.I.	p value
FVC(L)	Post	4.38	0,08	0,23	0,13	(0,05 − 0,11)	< 0,05
	Pre	4.46					
FEV1 (L)	Post	3.45	0,08	0,20	0,11	(0,05 − 0,09)	< 0,05
	Pre	3.53					
FEV1/FVC(%)	Post	78.76	0,44	4,79	0,26	(0,09–0,96)	0,10
	Pre	79.19					
PEF (L/s)	Post	8.43	0,15	1,07	0,06	(0,03–0,26)	< 0,05
	Pre	8.58					

S.D. = Standard Deviation, S.E. = Standard Error, C.I. = Confidence Interval

Subsequently, we tested the correlation between the COVID-19 and the worsening of each lung function parameter. As the different strains of SARS-CoV-2 showed different pathogenicity in the upper and lower respiratory tract, we analyzed the influence of infections in the three study years (2020, 2021 and 2022) separately. Linear regression showed a statistically significant difference in FVC between infected and non-infected subjects in 2020 and 2021, but no significant difference in 2022, as shown in Table 4.

**Table 4:** Main lung function parameters compared with year of COVID-19

	2020		2021		2022	
	p	S.E.	p	S.E.	p	S.E.
Decline in FVC	< 0,05	0,040	< 0,05	0,053	0,880	0,026
Decline in FEV1	0,104	0,036	0,210	0,047	0,339	0,023
Decline in FEV1/FVC	0,641	0,828	0,325	1,081	0,877	0,538
Decline in PEF	0,754	0,185	0,440	0,242	0,905	0,120

S.E. = Standard Error

To assess the protective effect of SARS-CoV-2 vaccination on respiratory function, we performed a linear regression between the examined variables (FVC, FEV1, Tiffenau index and PEF expressed as a percentage reduction in mL compared to the period before the pandemic) and the investigated factors (age, BMI, gender, current smoking habit, SARS-CoV-2 infection, prior vaccination, number of infections) for multivariate analysis. The decline in FVC was significantly lower in subjects who had received vaccination prior to infection. Results are shown in Table 5.

**Table 5:** Linear regression analysis

**Decline in FVC**			
	B	S. E.	p value
Age	0.004	0.001	< 0.05
BMI	0.005	0.003	0.121
Gender	0.017	0.027	0.523
Smokers	-0.015	0.016	0.347
COVID-19	0.049	0.077	0.528
Vaccination before infection	-0.079	0.041	< 0.05
Number of infection	0.043	0.049	0.383
**Decline in FEV1**			
	B	S. E.	p value
Age	0.000	0.001	0.709
BMI	0.000	0.003	0.940
Gender	0.043	0.025	0.084
Smokers	0.000	0.014	0.979
COVID-19	0.058	0.070	0.408
Vaccination before infection	-0.044	0.037	0.230
Number of infection	0.018	0.045	0.688
**Decline in PEF**			
	B	S. E.	p value
Age	0.014	0.006	< 0.05
BMI	-0.010	0.015	0.512
Gender	0.620	0.123	< 0.05
Smokers	-0.079	0.071	0.268
COVID-19	0.342	0.348	0.326
Vaccination before infection	0.041	0.183	0.825
Number of infection	-0.154	0.222	0.489
**Decline in FVC/FEV1**			
	B	S. E.	p value
Age	-0.030	0.026	0.248
BMI	-0.076	0.070	0.278
Gender	0.137	0.568	0.809
Smokers	0.816	0.328	< 0.05
COVID-19	-0.089	1.605	0.956
Vaccination before infection	0.512	0.845	0.545
Number of infection	-0.486	1.024	0.635

S.E. = Standard Error

## Discussion

It is now more than evident that COVID-19 is a complex disease, capable of manifesting itself in a rather varied manner in the population, affecting many organs and systems with varying degrees of severity. The importance of our study lies in the inclusion of individuals without significant comorbidities who showed a statistically significant decline in lung function indices (FVC, FEV1) during follow-up spirometry, which persisted over the medium to long term.

Our study revealed subclinical changes in the lung function of the subjects included in the sample. Since a physiological decline in lung function indices is expected over a three-year period, in order to highlight the role of COVID-19 infection, we compared the loss of FEV1, FVC and FEV1/FVC in the group of subjects who had the infection in comparison to those who did not become infected. Although no significant differences were found between the two groups overall when considering COVID-19 infection as a whole, the analysis of the different years showed that infection with in 2020 and 2021 (time of spread of the first variant strains) was associated with a slight but significant reduction in FVC. Previous studies had already found a reduction in lung function indices in patients with COVID-19. [10]

Referring to another international study, the COMMUNITY study assessed long-term immunity in healthcare workers at a Swedish hospital who had a mild form of the disease during the acute phase [11]. The study had an 8-month follow-up until early 2021. Participants reported symptoms through questionnaires and had periodic antibody titer evaluations through blood tests. Results showed that many low-risk subjects with mild SARS-CoV-2 infection experienced diverse long-term symptoms, negatively impacting their lives. However, the analysis has limitations, such as relying on subjective symptom evaluation and omitting objective data like spirometric indices. Nevertheless, this adds to the hypothesis that COVID-19 is not solely an acute disease, and our study, involving young and healthy individuals with a history of mild acute disease, supports the theory of Long COVID possibility.

To test the hypothesis that previous vaccination with Comirnaty (BNT162b2) is protective with respect to worsening lung function indices, we entered this vaccination into a multiple regression model, considering age, gender, BMI and smoking as covariates. Vaccination was statistically associated with a lower loss of FVC emphasising the importance of vaccination in protecting even from subclinical effects of COVID-19 infection.

It is necessary to specify that our investigation was primarily limited by the relatively small sample size (321 operators), which could have led to falsely negative results in determining additional variables of functional loss. However, given the correlation between SARS-CoV-2 positivity and changes in functional indices, it is reasonable to assume that this result was not influenced by experimental limitations.

There is a notable heterogeneity in the number of COVID-19 cases that developed within our sample over different years. This is another limitation of our study, attributable to the epidemiological characteristics of the infection.

Certainly, further studies are required to extend the follow-up period, allowing for a better evaluation of the significance of the reductions in spirometric indices recorded by us, which are still in the subclinical phase. As a result, the prognostic significance of our analysis results remains limited.

Undoubtedly, the evidence of unfavorable functional outcomes in a population represented by subjects without risk factors and with a history of mild disease can contribute to increasing the perception of COVID-19-related risk. The lack of risk perception resulting from the disease has been responsible for a significant increase in vaccine hesitancy, even among healthcare workers.

Despite the aforementioned limitations, our work demonstrates the effectiveness of the vaccination campaign in preventing unfavorable outcomes on functional indices, a factor to consider in defining vaccination strategies for populations at higher risk of infection, such as healthcare workers.

The results of our study shed important light on the impact of subclinical infection in 2020 and 2021, which coincides with the circulation of the alpha, beta and delta variants of SARS-CoV-2. Notably, we observed that such subclinical infections were associated with a deterioration of key respiratory parameters, specifically FVC and FEV1. This suggests that even mild or asymptomatic infections during the delta variant-dominated period could have significant implications for respiratory health.

On a positive note, our research also revealed a promising trend concerning vaccination. We found that individuals who received vaccination appeared to be better protected against the adverse respiratory outcomes associated with subclinical infections. Even in healthy subjects who had experienced an infection, the presence of vaccination seemed to exert a protective effect on respiratory function, potentially mitigating the impact of the infection on lung health. In the literature, we can see that vaccination is associated with a reduction in inflammation in the short and long term, which could partly explain the reduced impact of COVID-19 on lung function parameters in vaccinated subjects [12].

These findings underscore the crucial importance of widespread vaccination, especially among healthcare workers. As frontline heroes battling the COVID-19 pandemic, their health and well-being are of paramount importance. By ensuring universal vaccination of healthcare workers against COVID-19, we can take proactive measures to safeguard their respiratory health and reduce the risk of potential long-term consequences resulting from subclinical infections.

In summary, our study not only emphasizes the significance of addressing subclinical infections during the delta variant surge but also highlights the encouraging role of vaccination in mitigating the adverse respiratory effects of such infections. Implementing universal vaccination among healthcare workers is a vital step towards safeguarding their health and the well-being of the broader community in the ongoing fight against the COVID-19 pandemic.

## Conclusion

In conclusion, our study shows that subclinical infection during the year 2020 and 2021 is associated with a worsening of respiratory parameters (FVC and FEV1), but vaccination appears to be associated with protection against these outcomes.

In addition, it is highlighted that even in healthy subjects who have had an infection, respiratory outcomes can be detected, towards which vaccination appears to have a protective effect, shown in all lung function parameters, but particularly in the decrease in FVC. This evidence underlines the need for universal vaccination of healthcare workers against COVID-19.

## Data Availability

Datasets used and/or analyzed during the current study are available from the corresponding author on reasonable request.
